# OsbHLH058 and OsbHLH059 transcription factors positively regulate iron deficiency responses in rice

**DOI:** 10.1007/s11103-019-00917-8

**Published:** 2019-09-24

**Authors:** Takanori Kobayashi, Asami Ozu, Subaru Kobayashi, Gynheung An, Jong-Seong Jeon, Naoko K. Nishizawa

**Affiliations:** 1 Research Institute for Bioresources and Biotechnology, Ishikawa Prefectural University, 1-308 Suematsu, Nonoichi, Ishikawa 921-8836 Japan; 2 Crop Biotech Institute and Graduate School of Biotechnology, Kyung Hee University, Yongin, 17104 Korea; 3 Graduate School of Agricultural and Life Sciences, The University of Tokyo, 1-1-1 Yayoi, Bunkyo-ku, Tokyo, 113-8657 Japan

**Keywords:** bHLH transcription factor, HRZ ubiquitin ligase, Iron deficiency, Rice

## Abstract

**Key message:**

Subgroup IVc basic helix-loop-helix transcription factors OsbHLH058 and OsbHLH059 positively regulate major iron deficiency responses in rice in a similar but distinct manner, putatively under partial control by OsHRZs.

**Abstract:**

Under low iron availability, plants transcriptionally induce the expression of genes involved in iron uptake and translocation. OsHRZ1 and OsHRZ2 ubiquitin ligases negatively regulate this iron deficiency response in rice. The basic helix-loop-helix (bHLH) transcription factor OsbHLH060 interacts with OsHRZ1, and positively regulates iron deficiency-inducible genes. However, the functions of three other subgroup IVc bHLH transcription factors in rice, OsbHLH057, OsbHLH058, and OsbHLH059, have not yet been characterized. In the present study, we investigated the functions of OsbHLH058 and OsbHLH059 related to iron deficiency response. *OsbHLH058* expression was repressed under iron deficiency, whereas the expression of *OsbHLH057* and *OsbHLH060* was moderately induced. Yeast two-hybrid analysis indicated that OsbHLH058 interacts with OsHRZ1 and OsHRZ2 more strongly than OsbHLH060, whereas OsbHLH059 showed no interaction. An in vitro ubiquitination assay detected no OsbHLH058 and OsbHLH060 ubiquitination by OsHRZ1 and OsHRZ2. Transgenic rice lines overexpressing *OsbHLH058* showed tolerance for iron deficiency and higher iron concentration in seeds. These lines also showed enhanced expression of many iron deficiency-inducible genes involved in iron uptake and translocation under iron-sufficient conditions. Conversely, *OsbHLH058* knockdown lines showed susceptibility to iron deficiency and reduced expression of many iron deficiency-inducible genes. *OsbHLH059* knockdown lines were also susceptible to iron deficiency, and formed characteristic brownish regions in iron-deficient new leaves. *OsbHLH059* knockdown lines also showed reduced expression of many iron deficiency-inducible genes. These results indicate that OsbHLH058 and OsbHLH059 positively regulate major iron deficiency responses in a similar but distinct manner, and that this function may be partially controlled by OsHRZs.

**Electronic supplementary material:**

The online version of this article (10.1007/s11103-019-00917-8) contains supplementary material, which is available to authorized users.

## Introduction

Iron (Fe) is an essential element for living organisms, functioning as an essential component of prosthetic groups containing heme and Fe-sulfur clusters, and also as free Fe ions that bind to proteins for specific functions. Both Fe uptake and internal translocation are crucial for Fe homeostasis in multicellular organisms, including animals and plants. Plants take up Fe from the rhizosphere, where Fe is abundant but only slightly soluble. Fe solubility and, thus, availability is especially low in aerobic and high-pH soils, including calcareous soils, which cover one-third of cultivated areas globally, severely limiting plant productivity and nutritional quality (Marschner 1995; Briat et al. 2015). Low Fe content in staple foods such as polished rice (*Oryza sativa* L.) is also a main cause of human anemia, which affects at least 2 billion people worldwide (Murgia et al. 2012; Briat et al. 2015). Therefore, understanding the mechanisms of plant Fe uptake and translocation is important for improving plant productivity in problem soils and Fe nutritional quality for the human diet.

Graminaceous plants such as rice and barley utilize a unique Fe uptake system characterized by the production of high-affinity ferric Fe [Fe(III)] chelators, which are designated mugineic acid family phytosiderophores (MAs); these solubilize Fe(III) in the rhizosphere, and the resulting Fe(III)-MA complexes are taken up by roots (Takagi 1976). In contrast, non-graminaceous plants rely on the reduction of rhizospheric Fe(III) and subsequent uptake of ferrous Fe ion (Fe^2+^) for Fe uptake (Römheld and Marschner 1986). Fe^2+^ uptake also occurs in graminaceous species such as rice (Ishimaru et al. 2006). Various types of chelators and transporters also play essential roles in Fe translocation within the plant body (Kobayashi and Nishizawa 2012; Kobayashi et al. 2019).

In response to low iron availability, plants induce the expression of a set of genes involved in Fe uptake and translocation. This response is mediated at the transcript level by cascades comprising transcription factors (TFs), and modulated by ubiquitin ligases (Kobayashi and Nishizawa 2012; Gao et al. 2019; Kobayashi 2019). This regulation has mainly been investigated in rice and *Arabidopsis thaliana*. Irrespective of clear divergences in Fe uptake systems between rice and *Arabidopsis*, many regulatory components of TFs and ubiquitin ligases are conserved regarding similarities in function and sequence (Kobayashi 2019). The conserved TFs belong to the Ib, IVb, and IVc subgroups of the basic helix-loop-helix (bHLH) family (Gao et al. 2019; Kobayashi 2019). The bHLH TFs are widely distributed among eukaryotic organisms, among which higher plants possess especially large numbers; for example, rice and *Arabidopsis* have 173 and 158 bHLH proteins, respectively (Pires and Dolan 2010). Plant bHLH proteins can be phylogenetically divided into 26 subfamilies (Pires and Dolan 2010). The formation of homodimers or heterodimers is a prerequisite for DNA binding and thus transcriptional regulation of bHLH TFs.

Subgroup Ib bHLH TFs, including rice OsIRO2 and *Arabidopsis* AtbHLH38, AtbHLH39, AtbHLH100, and AtbHLH101, induce the expression of large subsets of genes involved in Fe uptake and translocation (Ogo et al. 2007, 2011; Wang et al. 2007, 2013; Yuan et al. 2008; Sivitz et al. 2012). In contrast, subgroup IVb bHLH TFs, including rice OsIRO3 and *Arabidopsis* PYE and AtbHLH11, repress the expression of subsets of genes involved in Fe uptake and translocation (Long et al. 2010; Zheng et al. 2010; Tanabe et al. 2019). The expression of these subgroup Ib and IVb bHLH genes, except for *AtbHLH11*, is strongly induced under Fe deficiency at the transcript level (Ogo et al. 2006; Long et al. 2010; Zheng et al. 2010), indicating the presence of upstream TFs. Rice IDEF1, which belongs to ABI3/VP1 but is not a bHLH family TF, induces the expression of *OsIRO2* and *OsIRO3* (Kobayashi et al. 2007, 2009, 2014); however, *Arabidopsis* does not possess an apparent IDEF1 ortholog. *Arabidopsis* Ib bHLH TFs form heterodimers with FIT, a subgroup IIIa bHLH TF, to induce Fe uptake-related genes (Yuan et al. 2008; Wang et al. 2013), whereas rice lacks an apparent FIT ortholog, and OsIRO2 does not appear to require heterodimer counterparts for its function.

Recent studies have clarified that some subgroup IVc bHLH TFs, including rice OsbHLH060 (also designated OsPRI1) and *Arabidopsis* AtbHLH34, AtbHLH104, AtbHLH105 (also designated ILR3), and AtbHLH115, act upstream of subgroup Ib and IVb bHLH TFs by inducing their transcriptional expression (Selote et al. 2015; Zhang et al. 2015; Li et al. 2016; Liang et al. 2017; Zhang et al. 2017). The transcript levels of these *subgroup IVc bHLHs* are affected little by Fe nutritional status (Zhang et al. 2015; Liang et al. 2017; Zhang et al. 2017). *Arabidopsis* subgroup IVc bHLH TFs interact with each other and with PYE (Long et al. 2010; Selote et al. 2015; Zhang et al. 2015; Li et al. 2016; Liang et al. 2017), suggesting transcriptional regulation diversity through variation among heterodimers.

The expression of these Fe deficiency-inducible genes involved in Fe uptake and translocation is negatively regulated by HRZ/BTS ubiquitin ligases at the transcript level (Kobayashi et al. 2013; Selote et al. 2015; Hindt et al. 2017). HRZ/BTS proteins share conserved domain structures including hemerythrin, CHY, CTCHY and RING zinc (Zn)-fingers, and rubredoxin-type fold (Urzica et al. 2012; Kobayashi et al. 2013). The hemerythrin domain binds Fe and Zn, putatively participating in intracellular Fe sensing, analogous to mammal Fe sensor protein FBXL5 (Hindt and Guerinot 2012; Kobayashi et al. 2013; Selote et al. 2015; Rodríguez-Celma et al. 2019a). The RING Zn-finger usually binds Zn and serves as a component of E3 ubiquitin ligase, which ubiquitinates specific proteins for 26S proteasome-mediated degradation or modification (Hua and Vierstra 2011). This domain in *Arabidopsis* BTS is crucial for both its E3 ligase activity in vitro and its function in vivo (Selote et al. 2015; Matthiadis and Long 2016; Hindt et al. 2017). CHY and CTCHY Zn-fingers are also predicted to bind Zn and putatively mediate transcriptional, post-transcriptional, or post-translational gene regulation (Gamsjaeger et al. 2007; Sheng et al. 2008). The rubredoxin-type fold (also designated the Zn ribbon) is predicted to bind Fe or Zn, forming a Fe–sulfur cluster when bound to Fe (Sieker et al. 1986; Sheng et al. 2008).

Knockdown of one or both of the rice *HRZ* genes, *OsHRZ1* and *OsHRZ2*, results in markedly enhanced transcript expression of most of the Fe deficiency-inducible genes involved in Fe uptake and translocation, tolerance to low Fe availability, hypersensitivity to extreme Fe excess conditions, and Fe accumulation in shoots and seeds (Kobayashi et al. 2013; Aung et al. 2018). Similar phenotypes are also observed in knockout mutants of *OsHRZ1* (Zhang et al. 2017) and knockdown and point mutants of *Arabidopsis BTS* (Long et al. 2010; Selote et al. 2015; Hindt et al. 2017), a functional ortholog of rice *OsHRZ1* and *OsHRZ2*. Transcript levels of *OsHRZ1*, *OsHRZ2*, and *BTS* are relatively high in shoots and root steles, and are induced under Fe deficiency (Long et al. 2010; Kobayashi et al. 2013). In addition, BTS protein accumulation is increased under lower Fe abundance in in vitro translation reactions (Selote et al. 2015). However, physiological analyses suggested that HRZ/BTS regulatory activity increase with Fe abundance (Kobayashi et al. 2013; Hindt et al. 2017; Aung et al. 2018). This Fe-dependent activity of HRZ/BTS may be important for inhibiting excessive Fe uptake and translocation, thus avoiding Fe toxicity. *Arabidopsis* possesses two more HRZ/BTS homologs, BTSL1 and BTSL2, which have been suggested to play similar but distinct roles, mainly functioning in the root epidermis and cortex (Hindt et al. 2017; Rodríguez-Celma et al. 2019b).

Recent studies have identified some members of subgroup IVc bHLHs as putative ubiquitination targets of HRZ/BTS through the investigation of protein binding, in vivo protein expression, ubiquitination, or degradation assays (Selote et al. 2015, 2018; Zhang et al. 2017). Rice OsbHLH060 interacts with OsHRZ1; the latter is thought to ubiquitinate the former for degradation (Zhang et al. 2017). Genetic studies have indicated that the Fe deficiency tolerance and enhanced expression of Fe-related genes observed in an *OsHRZ1* knockout line is partly dependent on the presence of *OsbHLH060* (Zhang et al. 2017). Similarly, *Arabidopsis* AtbHLH105 and AtbHLH115 interact with BTS, and the abundance of these IVc bHLH proteins is negatively regulated by BTS (Selote et al. 2015), suggesting ubiquitination-dependent degradation. AtbHLH104 also interacts with BTS; however, the protein level of AtbHLH104 is apparently not regulated by BTS (Selote et al. 2015). Two NAC-family TFs, VOZ1 and VOZ2, may also be degraded via BTS-dependent ubiquitination (Selote et al. 2018). Although VOZ1 and VOZ2 have no apparent function related to Fe deficiency tolerance, decreased abundance of the VOZ2 protein through *BTS* overexpression results in tolerance to drought stress in *Arabidopsis* (Selote et al. 2018). A similar approach showed that FIT, a master regulator in *Arabidopsis* Fe deficiency response (Colangelo and Guerinot 2004; Jakoby et al. 2004), is an ubiquitination target of BTSL1 and BTSL2 (Rodríguez-Celma et al. 2019b).

These findings regarding bHLH TFs and HRZ/BTS ubiquitin ligases suggest the presence of a general regulatory pathway of HRZ/BTS → IVc bHLHs → Ib/IVb bHLHs → Fe deficiency-inducible genes (Online Resource Fig. S1a; Gao et al. 2019; Kobayashi 2019). However, it remains an open question whether this pathway fully explains the wide-ranging inhibitory functions of HRZ/BTS, due to a lack of comprehensive studies on ubiquitination substrates of HRZ/BTS and regulatory targets of all IVc bHLH TFs. Although OsbHLH060 and all four IVc bHLH TFs in *Arabidopsis* induce both Ib and IVb bHLH genes (Zhang et al. 2015; Li et al. 2016; Liang et al. 2017; Zhang et al. 2017), it is unknown how the balance between gene induction by Ib bHLHs and repression by IVb bHLHs could coordinate to produce proper Fe deficiency responses. In addition, IVc bHLHs may directly regulate some Fe deficiency-inducible genes without mediating Ib and IVb bHLHs; however, this possibility has not yet been explored. Moreover, rice possesses three other *subgroup IVc bHLH* genes in addition to *OsbHLH060*, namely *OsbHLH057*, *OsbHLH058*, and *OsbHLH059* (Zheng et al. 2010), but their functions have yet to be investigated. To date, there has been no report of possible ubiquitination targets of OsHRZ2, except for self-ubiquitination activity in OsHRZ2 as well as OsHRZ1 (Kobayashi et al. 2013). Therefore, the objective of the present study was to characterize OsbHLH058 and OsbHLH059 functions related to Fe deficiency responses involving HRZ ubiquitin ligases. We show that OsbHLH058 and OsbHLH060, but not OsbHLH059, interact with OsHRZ1 and OsHRZ2. Our results further indicate that OsbHLH058 and OsbHLH059 positively regulate major iron deficiency responses in rice (Fig. S1b).

## Materials and methods

### Plant materials and growth conditions

We used wild-type rice (*O. sativa* L.) cultivar Nipponbare, cultured for a previous study (Kobayashi et al. 2013) for *OsbHLH* gene expression analysis. Transgenic lines possessing *OsbHLH* genes were obtained as described below. T-DNA insertion lines *b059*-*1* (1B-08437) and b059OX-1 (3A-09300) (background cultivar: Dongjin) were obtained from the Rice T-DNA Insertion Sequence Database (POSTECH; Pohang University of Science and Technology, Pohang, Korea; Jeong et al. 2002).

Seeds were germinated on Murashige and Skoog (MS) medium (Murashige and Skoog 1962) with or without hygromycin B (50 mg L^−1^) for transformants and non-transformants (NT), respectively. After 18–22 d of culture at 28 °C under a 14-h light/10-h dark cycle followed by 3 d of acclimation, plantlets were transferred to a hydroponic solution in a greenhouse under a 30 °C light/25 °C dark cycle with natural light conditions. The composition of the hydroponic solution was as follows: 0.70 mM K_2_SO_4_, 0.10 mM KCl, 0.10 mM KH_2_PO_4_, 2.0 mM Ca(NO_3_)_2_, 0.50 mM MgSO_4_, 10 μM H_3_BO_3_, 0.50 μM MnSO_4_, 0.50 μM ZnSO_4_, 0.20 μM CuSO_4_, 0.01 μM (NH_4_)_6_Mo_7_O_24_, and 100 μM Fe(III)–EDTA. After 5–6 days of preculture in hydroponic solution, Fe deficiency was initiated by omitting Fe(III)-EDTA from the solution. The nutrient solution was renewed after 4 days of Fe deficiency and sufficiency (control) treatments. After 7–9 days, whole roots and leaf blades were harvested from three plants for each replicate, and immediately frozen in liquid nitrogen for expression analysis, or cut into pieces and immediately dried for metal concentration analysis.

Pot cultivation was conducted using normal soil by transplanting each seedling into a 1-L pot filled with a 1:1 mixture of Bonsol (Sumitomo Chemical, Japan) and vermiculite (Protoleaf, Japan), supplied with 0.5 g each of EcoLongTotal 70 and LongTotal 140 controlled-release fertilizers (JCAM AGRI, Japan) until seed maturation.

### Quantitative reverse-transcription polymerase chain reaction (qRT-PCR) analysis

RNA extraction, DNase treatment, reverse-transcription and qRT-PCR were performed as described in Kobayashi et al. (2016), using primers listed in Online Resource Table S1. Transcript abundance was expressed as a ratio relative to the levels in Fe-sufficient NT roots in each experiment.

### Yeast two-hybrid assay

To construct *OsHRZ1* and *OsHRZ2* bait vectors, the RING Zn-finger domain was deleted by inverse PCR using primers listed in Table S1 and pENTR/D-TOPO-*OsHRZ* vectors (Kobayashi et al. 2013) as templates. For the HRZ1ΔHrRi, HRZ1ΔHrRiRub and HRZ1ΔHrRiZnF baits, OsHRZ12-delRING-F and OsHRZ1-delRING-R were used as the primers and the pENTR/D-TOPO-OsHRZ1 ΔH vector was used as a template. For the HRZ2ΔRi bait, OsHRZ12-delRING-F and OsHRZ2-delRING-R were used as the primers and the pENTR/D-TOPO-OsHRZ2 FL vector was used as a template. Amplified fragments were purified and self-ligated, and the sequences were verified. The resultant plasmids with expected RING Zn-finger deletions of amino acids 1112–1180 for OsHRZ1 and 687–755 for OsHRZ2 in frame were used for the HRZ1ΔHrRi and HRZ2ΔRi baits, respectively. Using the same method, plasmids with spontaneous OsHRZ1 deletions of amino acids 1109–1212 and 888–1180 in frame were also obtained; these were used for HRZ1ΔHrRiRub and HRZ1ΔHrRiZnF baits, respectively. The *HRZ* inserts of these plasmids were excised using *Eco*RI and *Sal*I, and inserted into pGBKT7 (TaKaRa, Japan) at the same sites. To construct HRZ1Hr baits, the plasmid pENTR/D-TOPO-OsHRZ1 ΔRZ (Kobayashi et al. 2013) was digested with *Nco*I, and the excised partial insert corresponding to OsHRZ1 amino acids 7–456 was subcloned into pGBKT7 at the same site.

Full-length open reading frames of *OsbHLH057*, *OsbHLH058*, *OsbHLH059*, and *OsbHLH060* were amplified by PCR using primers listed in Table S1 and a complementary DNA (cDNA) pool of Nipponbare rice cultivar roots and leaves (Kobayashi et al. 2013), and cloned into pENTR/D-TOPO (Invitrogen, USA); the sequences were then verified. Fragments were excised at the primer sites using restriction enzymes *Eco*RI and *Sal*I for *OsbHLH057*, or *Eco*RI and *Bam*HI for *OsbHLH058*, *OsbHLH059*, and *OsbHLH060*, and inserted into pGADT7AD (TaKaRa) at the same sites.

A yeast two-hybrid assay was performed using the Matchmaker Gold Yeast Two-Hybrid System (TaKaRa) following the manufacturer’s protocol (PT4084-1). Briefly, bait and prey vectors were separately transformed into yeast (*Saccharomyces cerevisiae*) strains Y2HGold and Y187, respectively, and grown on Synthetic Defined (SD) medium without tryptophan and leucine (SD-W and SD-L), respectively. Control bait plasmid pGBKT7-53, which expresses the GAL4 DNA-binding domain (BD) fused with murine p53, and prey plasmid pGADT7-T, which expresses the GAL4 activation domain (AD) fused with SV40 large T-antigen, supplied by the manufacturer, were also transformed. Bait–prey combinations were then produced by mating on SD-LW medium, and growth was tested by spotting 10 μL of each culture adjusted to OD_600_ = 1.0 onto SD selective media without leucine, tryptophan, histidine and adenine (–L–W–H–A), that without leucine, tryptophan and histidine (–L–W–H), and that without leucine and tryptophan supplemented with 5-bromo-4-chloro-3-indolyl-α-D-galactopyranoside (–L–W + X-α-Gal), followed by culture for 5 days at 30 °C. For further experiments (Fig. S3), bait–prey mated colonies were applied on SD selective medium –L–W–H–A + Aureobasidin A (AbA) or control medium SD–L–W, and grown for 5 days at 30 °C.

### In vitro ubiquitination assay

To produce recombinant proteins, *OsbHLH* gene fragments were excised from the abovementioned pENTR/D-TOPO vectors using *Eco*RI and *Pst*I for *OsbHLH058* and *OsbHLH060*, or *Eco*RI and *Sal*I for *OsbHLH059*, and inserted into pET42a(+) (Novagen, USA) at the same sites, constructing the *OsbHLH* in-frame downstream of glutathione-S-transferase (GST)-tag-His-tag-S-tag. These pET42a(+)-OsbHLH plasmids, as well as pET42a(+), which expresses GST-tag-His-tag-S-tag protein, as a negative control, were introduced into *Escherichia coli* strain BL21(DE3)pLysS. Recombinant proteins were obtained and purified using the GST fusion system (Novagen) according to the manufacturer’s instructions, except that *E*. *coli* was cultured at 16 °C. Purified proteins were concentrated using a VivaSpin 2-30 K column (GE Healthcare, USA).

Maltose binding protein (MBP)-fused OsHRZ proteins and MBP-LacZ control were prepared, and a ubiquitination assay conducted, as described in Kobayashi et al. (2013) using the Ubiquitinylation Kit (ENZO Life Sciences, USA), except that the potential GST-fused protein substrate (300 nM each) was added to the reaction. GST- and MBP-fused proteins were detected using an anti-GST primary antibody (MBL, Japan) and an anti-MBP primary antibody (Sigma, USA), respectively, at 1:1500 dilution in Can Get Signal solution 1 (Toyobo), followed by horseradish peroxidase-conjugated anti-mouse secondary antibody (GE Healthcare) at 1:10000 dilution in Can Get Signal solution 2 (Toyobo).

### Production of transgenic rice

To produce rice overexpression lines, the LR Clonase reaction (Invitrogen) was conducted using pH7FWG2 (Karimi et al. 2002) as the destination vector and abovementioned *OsbHLH058*-pENTR/D-TOPO as the entry vector, constructing in-frame fusion of *OsbHLH058* downstream of the cauliflower mosaic virus *35S* promoter. To produce knockdown rice lines by RNA interference, a 295-bp fragment corresponding to the 3′-untranslated region (3′UTR) and flanking coding region of *OsbHLH058*, or a 292-bp fragment corresponding to the 3′UTR of *OsbHLH059*, was amplified by PCR using primers listed in Table S1 and a cDNA pool of Tsukinohikari rice cultivar roots and leaves (Kobayashi et al. 2013) and inserted into pENTR/D-TOPO; the sequence was then verified. Using the LR Clonase reaction, the fragment was transferred into the destination vector, pIG121-RNAi-DEST (Ogo et al. 2007), to construct the RNA interference vector. Transformation of rice cultivar Tsukinohikari was performed as previously described (Hiei et al. 1994; Kobayashi et al. 2001), and T_1_ seeds were used for further analysis.

### Metal concentration analysis

Leaf segments and T_1_ brown seeds were dried for 2–3 days at 70 °C, and portions weighing 80–200 mg were wet-ashed with 1.5 mL 13.4 M HNO_3_ and 1.5 mL 8.8 M H_2_O_2_ for 20 min at 220 °C using a MarsXpress oven. Fe, Zn, Mn, and Cu concentrations were measured by inductively coupled plasma optical emission spectrometry (ICPS-8100; Shimadzu, Japan).

## Results

### Each of the four *subgroup IVc bHLH* genes show a distinct expression pattern

**Fig. 1:**
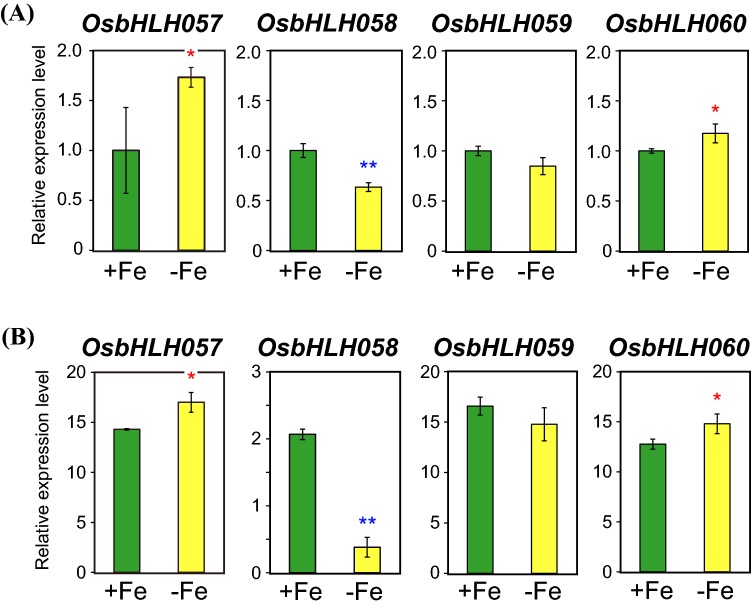
Expression levels of *subgroup IVc bHLH* genes. The transcript levels of *OsbHLH057*, *OsbHLH058*, *OsbHLH059*, and *OsbHLH060* were quantified by reverse-transcription polymerase chain reaction (RT-PCR) in roots (**a**) and leaves (**b**) of wild-type rice cultured under Fe sufficiency (+Fe) or deficiency (−Fe) for 7 days. Transcript abundance was normalized against the rice α-2 tubulin transcript level and expressed as a ratio relative to levels in +Fe roots. Means ± standard deviation (SD; n = 3) are shown. Asterisks indicate significant differences from the +Fe value (two-sample Student’s *t* test; *P < 0.05; **P < 0.01)

Amino acid sequences of the four rice subgroup IVc bHLH proteins, OsbHLH057, OsbHLH058, OsbHLH059, and OsbHLH060, are closely related (Fig. S2; Zheng et al. 2010). To estimate possible functions of each of these genes related to Fe deficiency responses in rice, we first examined the transcript levels of these genes in wild-type rice plants hydroponically cultured under Fe sufficiency and deficiency for 7 days by qRT-PCR (Fig. 1). In both roots and leaves, *OsbHLH057* and *OsbHLH060* expression was moderately induced under Fe deficiency (Fig. 1a, b). In contrast, *OsbHLH058* expression was moderately repressed in Fe-deficient roots and strongly repressed in Fe-deficient leaves (Fig. 1a, b). *OsbHLH059* expression was slightly repressed in Fe-deficient roots and leaves, although there were no significant differences in expression levels between Fe sufficiency and deficiency (Fig. 1a, b). *OsbHLH057*, *OsbHLH059*, and *OsbHLH060* expression levels were higher in leaves than in roots; however, that of *OsbHLH058* was similar between roots and leaves (Fig. 1a, b).

We also examined the expression pattern of these four genes using our previous 44 K microarray results (Ogo et al. 2008, 2014; Kobayashi et al. 2009, 2013; Table S2) and a publically available database. Fe deficiency responses in whole roots and shoots determined by microarray (Ogo et al. 2008) were similar to our qRT-PCR results (Fig. 1; Table S2). Tissue-specific microarray analysis after microdissection (Ogo et al. 2014) indicated that *OsbHLH058* expression was repressed in the root vascular bundle, cortex, and epidermis/exodermis under Fe deficiency, whereas *OsbHLH059* expression was repressed in the root cortex and epidermis/exodermis under Fe deficiency (Table S2). In contrast, *OsbHLH057* expression was induced in the root epidermis/exodermis under Fe deficiency (Table S2). Microarray results using *HRZ*, *IDEF1*, and *IDEF2* transformants (Kobayashi et al. 2009, 2013; Ogo et al. 2008) revealed that *OsbHLH057* expression was induced in both *OsHRZ* and *IDEF2* knockdown lines, but repressed in both *IDEF1* induction and *IDEF1* knockdown lines, whereas *OsbHLH058*, *OsbHLH059*, and *OsbHLH060* expression was little affected in these transformants (Table S2).

Inspection of the publicly available microarray database RiceXPro (Sato et al. 2011, 2013; http://ricexpro.dna.affrc.go.jp/index.html) revealed that all four *subgroup IVc bHLH* genes are widely expressed in various organs throughout the plant lifespan; however, some differences in expression patterns were observed. Notably, leaf expression of *OsbHLH058* and *OsbHLH060* showed diurnal rhythm with a steep peak at early daytime and late night-time, respectively, whereas diurnal alteration of *OsbHLH057* and *OsbHLH059* expression was less obvious. *OsbHLH058*, *OsbHLH059*, and *OsbHLH060* expression was induced in roots by jasmonic acid treatment, but *OsbHLH057* expression was not. Collectively, these results indicate that each of the *subgroup IVc bHLH* genes shows a distinct expression pattern at the transcript level. We mainly focused on *OsbHLH058* and *OsbHLH059* for further analysis, because expression pattern of these two genes in response to Fe deficiency was clearly distinct from that of previously characterized *OsbHLH060* (Fig. 1; Table S2).

### OsbHLH058 shows strong interaction with OsHRZs in yeast, but may not be ubiquitinated by OsHRZs

**Fig. 2:**
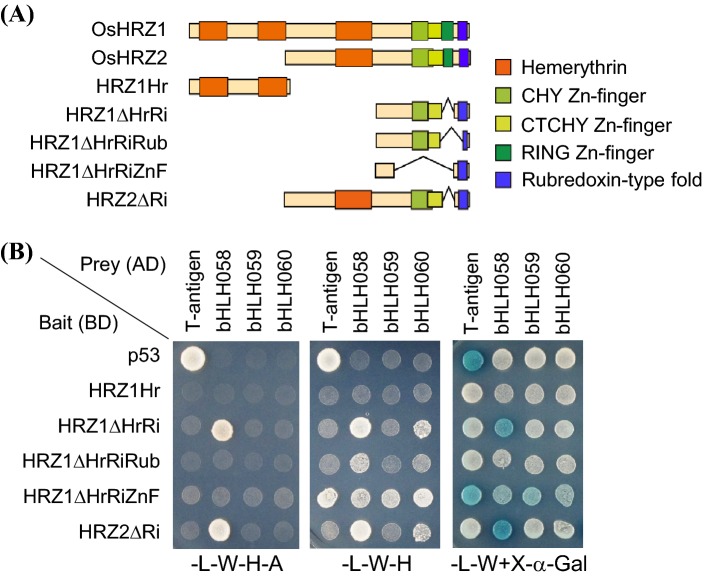
Yeast two-hybrid analysis of OsHRZ and subgroup IVc bHLH proteins. **a** Domain structures of OsHRZ partial sequences used for GAL4 DNA-binding domain (BD)-fusion. **b** Growth and color of yeasts transformed with BD-fused bait and GAL4 activation domain (AD)-fused prey after 5 days of culture. Interaction was detected by enhanced growth in high-stringency (–L–W–H–A) and low-stringency (–L–W–H) selective medium, or by blue staining in –L–W + X-α-Gal medium. Basal growth was confirmed in –L–W + X-α-Gal medium. BD-p53 and AD-large T antigen (T-antigen) are controls provided by the manufacturer

To explore possible relationships between subgroup IVc bHLH TFs and OsHRZs, we performed yeast two-hybrid analysis to examine interactions between these proteins. Because the RING Zn-finger domain of both OsHRZ1 and OsHRZ2 showed strong auto-activation activity when used as bait (data not shown), we used OsHRZ1 or OsHRZ2 lacking the RING Zn-finger domain, due to deletion, as a bait (Fig. 2a). For OsHRZ1, we used four partial sequences: an N-terminal fragment possessing the first two hemerythrin domains but no other domains (HRZ1Hr), a C-terminal fragment lacking the three hemerythrin domains and the RING Zn-finger domain (HRZ1ΔHrRi), a C-terminal fragment further lacking a major region of the rubredoxin-type fold and a minor part of the CTCHY Zn-finger domain due to deletion (HRZ1ΔHrRiRub), and a C-terminal fragment possessing the rubredoxin-type fold but lacking all three Zn-finger domains due to deletion (HRZ1ΔHrRiZnF). For OsHRZ2, we used a full-length sequence lacking only the RING Zn-finger domain due to deletion (HRZ2ΔRi). We also used p53 as a control bait. We used subgroup IVc bHLH TF or large T-antigen (T-antigen) as prey. As expected, T-antigen and p53 interacted with each other, as shown by superior growth in high-stringency and low-stringency selective media, –L-W–H–A and –L–W–H, respectively, as well as blue staining in –L–W + X-α-Gal medium (Fig. 2b). HRZ partial fragments did not show auto-activation activity when introduced with T-antigen control, except for mild auto-activation by HRZ1ΔHrRiZnF in –L–W–H and –L–W + X-α-Gal media (Fig. 2b).

In the high-stringency selective medium –L–W–H–A, OsbHLH058 clearly enhanced growth when introduced with HRZ1ΔHrRi or HRZ2ΔRi, but not with other baits analyzed (Fig. 2b). This result suggests that OsbHLH058 interacts with OsHRZ1 and OsHRZ2, in which the rubredoxin-type fold and the CHY or CTCHY Zn-finger or their N-terminal flanking region have essential roles, but hemerythrin and the RING Zn-finger domains are not required for interaction. In contrast, neither OsbHLH059 nor OsbHLH060 showed any interaction in –L–W–H–A medium (Fig. 2b). The same interaction pattern was also confirmed by blue staining in –L–W + X-α-Gal medium (Fig. 2b).

In the low-stringency selection medium –L–W–H, OsbHLH058 weakly enhanced growth with HRZ1ΔHrRiRub bait, in addition to strong growth activation with HRZ1ΔHrRi or HRZ2ΔRi (Fig. 2b). In this medium, mild growth activation was also observed by OsbHLH060 with HRZ1ΔHrRi or HRZ2ΔRi (Fig. 2b). This result suggests that OsbHLH060 interacts with OsHRZs in a manner similar to OsbHLH058 but with lower affinity. No interaction was observed for OsbHLH059, even in –L–W–H medium (Fig. 2b). We also performed a separate yeast two-hybrid analysis using OsbHLH057 or OsbHLH058 as prey. OsbHLH057 also showed no strong interaction with OsHRZs (Fig. S3). These results suggest that OsbHLH058 interacts with OsHRZ1 and OsHRZ2 more strongly than other subgroup IVc bHLH TFs.

**Fig. 3:**
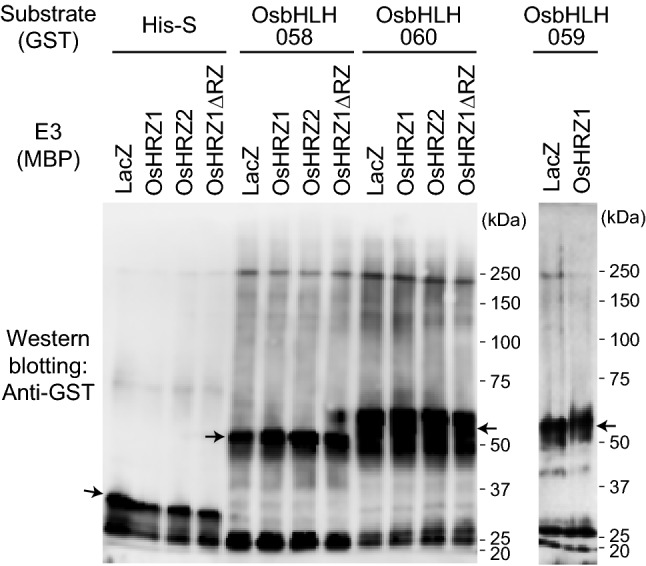
In vitro ubiquitination assay of OsbHLH058, OsbHLH060, and OsbHLH059 with OsHRZ ubiquitin ligases. Reaction was conducted with maltose binding protein (MBP) fusions of full-length OsHRZs as E3 ligases, and MBP-LacZ and MBP-OsHRZ1ΔRZ as negative controls, and GST fusions of OsbHLH058, OsbHLH060, and OsbHLH059 as potential substrates, or GST-tag-His-tag-S-tag (His-S) as a negative control. GST-fused proteins were detected by Western blotting using anti-GST antibody. The positions and sizes of the molecular mass markers are shown to the right of each blot. Monomeric free GST-fused proteins are indicated with arrows

We then tried to detect ubiquitination of subgroup IVc bHLH TFs by OsHRZs. We produced recombinant proteins of GST-fused OsbHLH058, OsbHLH059, and OsbHLH060, as well as GST-His-S as a negative control, and used these as potential substrates for our in vitro ubiquitination assay system (Kobayashi et al. 2013). We used MBP-fused full-length OsHRZ1 and OsHRZ2 as active E3 ubiquitin ligases, as well as MBP-LacZ and MBP-OsHRZ1ΔRZ, the latter of which lacks the RING and other Zn-finger domains and thus lacks E3 ubiquitin ligase activity (Kobayashi et al. 2013), as E3 negative controls. When we added either OsbHLH058 or OsbHLH060 to the reaction solution, high-molecular-weight smear bands appeared in all lanes, irrespective of presence or absence of E3 ligase activity, as determined by substrate detection using anti-GST antibody (Fig. 3). We did not observe more intense bands or smear staining of OsbHLH058 or OsbHLH060 with active OsHRZ1 or OsHRZ2. A similar result was obtained with OsbHLH059 with or without active OsHRZ1. E3 ligase activity in OsHRZ1 and OsHRZ2 was confirmed by appearance of much stronger high-molecular-weight smear bands than that in negative controls using anti-MBP antibody (Fig. S4; Kobayashi et al. 2013). These results indicate that OsbHLH058, OsbHLH059, and OsbHLH060 were not ubiquitinated by OsHRZ1 or OsHRZ2 in the experimental conditions of this study.

### *OsbHLH058* overexpression rice shows tolerance to Fe deficiency and enhanced expression of Fe deficiency-inducible genes

Although we did not detect ubiquitination of OsbHLH058 by OsHRZs, their protein interaction and Fe deficiency-repressed *OsbHLH058* expression suggest the possible involvement of OsbHLH058 in Fe responses and homeostasis. To address this possibility, we produced transgenic rice lines constitutively overexpressing *OsbHLH058* under the control of the cauliflower mosaic virus *35S* promoter (designated b058OX lines). We selected representative lines, b058OX-2 and -4, showing enhanced *OsbHLH058* expression (Fig. S5a), for further analysis.

**Fig. 4:**
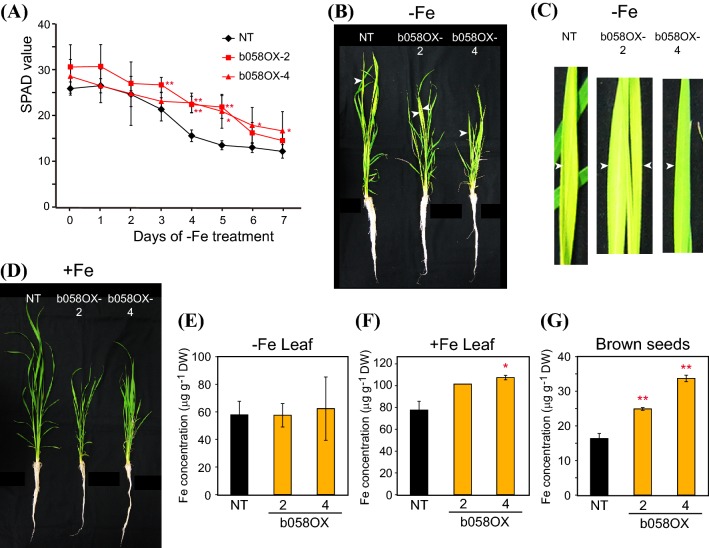
Fe deficiency tolerance of *OsbHLH058* overexpression plants. **a** Fe deficiency tolerance in hydroponic culture. Relative chlorophyll contents (SPAD values) of the newest leaves were measured after the onset of Fe deficiency treatment. **b** Representative plants on day 7 of Fe-deficient hydroponic culture. Three plants were bundled for culture of each line. **c** Representative newest leaves on day 7 of Fe-deficient hydroponic culture. White arrowheads in **b** and **c** indicate the same positions. **d** Representative plants on day 7 of Fe-sufficient hydroponic culture. Three plants were bundled for culture of each line. **e, f** Fe concentration in leaf blades after Fe-deficient (**e**) or -sufficient (**f**) hydroponic culture for 7 days. **g** Fe concentration in brown seeds after pot culture in Fe-sufficient soil. NT, non-transformant; b058OX-2 and 4, *OsbHLH058* overexpression lines 2 and 4, respectively. Means  ±  SD are shown (**a** n = 12 for NT, n = 5 for b058OX-2, n = 4 for b058OX-4; **e** n = 4 for NT, n = 2 for b058OX-2 and 4; **f** n = 3 for NT, n = 2 for b058OX-4; n = 1 for b058OX-2; **g** n = 7 for NT, n = 3 for b058OX-2 and 4). Asterisks indicate significant differences compared to NT at each time point and condition (two-sample Student’s *t*-test; *P < 0.05; **P < 0.01)

These lines and NT rice were cultured hydroponically and subjected to Fe deficiency treatment. The chlorophyll level in the newest leaf as determined by SPAD value, which is indicative of Fe nutrition, was higher in b058OX lines 2 and 4 than in NT especially during days 4–5 of Fe deficiency treatment (Fig. 4a). The newest leaves of b058OX line 4 still remained greener than those of NT after 7 days of Fe deficiency treatment (Fig. 4b, c). These results suggest that the b058OX lines are tolerant to low Fe availability. These lines and NT showed similar leaf colors under Fe sufficiency (Fig. 4d). The b058OX lines showed retarded growth from the beginning of seedling stages, which led to shorter shoot length until the end of hydroponic culture under either Fe condition (Fig. 4b, d). However, the growth rates were similar between the genotypes during the Fe treatments (data not shown).

We then measured Fe concentrations in the leaves of these plants. Both b058OX lines contained leaf Fe concentrations similar to NT levels after Fe-deficient hydroponic culture (Fig. 4e), but tended to contain higher leaf Fe concentration than NT after Fe-sufficient hydroponic culture (Fig. 4f). Both b058OX lines contained significantly higher Fe concentrations in brown seeds than NT after normal soil culture (Fig. 4g).

**Fig. 5:**
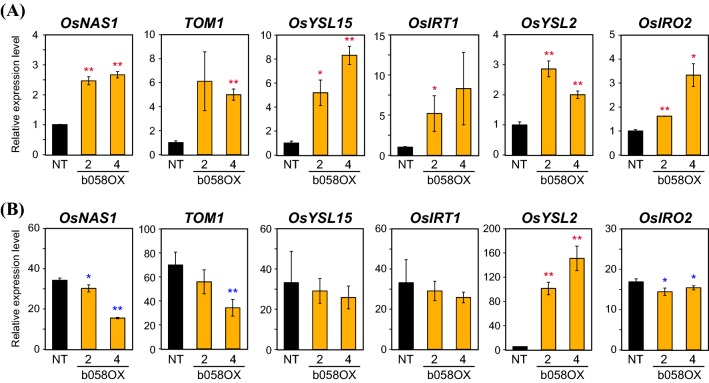
Expression levels of representative Fe-related genes in *OsbHLH058* overexpression plants. Transcript levels in roots of *OsbHLH058* overexpression (b058OX) lines 2 and 4 and non-transformants (NT) were quantified by RT-PCR, after 7 days of hydroponic culture under Fe sufficiency (**a**) or deficiency (**b**). Transcript abundance was normalized against the rice α-2 tubulin transcript level and expressed as a ratio relative to the levels in the +Fe NT roots. Means ± SD (n = 3) are shown. Asterisks indicate significant differences from the NT value at each condition (two-sample Student’s *t*-test; *P < 0.05; **P < 0.01)

We then investigated the expression patterns of Fe-related genes in roots of these b058OX lines after hydroponic culture. We quantified the transcript levels of six representative Fe deficiency-inducible genes: *OsNAS1*, *TOM1*, and *OsYSL15*, which are responsible for deoxymugineic acid (DMA)-based Fe(III) uptake and translocation, *OsIRT1* for Fe^2+^ uptake, *OsYSL2* for Fe(II)-nicotianamine transport for internal Fe translocation, and *OsIRO2* for positive regulation of genes involved in Fe(III) uptake and translocation (Kobayashi et al. 2014). Under Fe-sufficient conditions, the expression of all genes was markedly induced in the b058OX-2 and 4 lines compared with NT (Fig. 5a), suggesting positive regulation of these genes by OsbHLH058. Under Fe deficiency, however, these genes were not more highly expressed in b058OX-2 and 4 than in NT, except for still greater induction of *OsYSL2* (Fig. 5b). *OsNAS1*, *TOM1*, and *OsIRO2* expression was moderately lower in the b058OX-2 and 4 lines than in NT under Fe deficiency (Fig. 5b).

### Altered expression level of *OsbHLH059* influences rice Fe deficiency responses

**Fig. 6:**
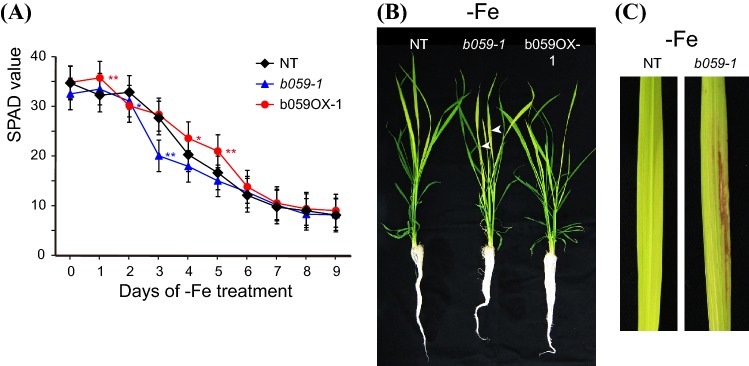
Fe deficiency tolerance of *OsbHLH059* T-DNA insertion lines. **a** Fe deficiency tolerance in hydroponic culture. Relative chlorophyll contents (SPAD values) of the newest leaves were measured after the onset of Fe deficiency treatment. **b** Representative plants on day 9 of Fe-deficient hydroponic culture. Three plants were bundled for culture of each line. White arrowheads indicate representative positions of brown spots in the newest leaves. **c** Representative newest leaves on day 9 of Fe-deficient hydroponic culture. NT, non-transformant; *b059*-*1*, *OsbHLH059* knockdown line by T-DNA insertion; b059OX-1, *OsbHLH059* overexpression line by T-DNA insertion. Means ± SD (n = 12) are shown for **a**. Asterisks indicate significant differences compared to NT (two-sample Student’s *t*-test; *P < 0.05; **P < 0.01)

To explore further the possible role of OsbHLH058 and OsbHLH059 in Fe nutrition, we then searched for T-DNA insertion rice lines from the POSTECH database (Jeong et al. 2002). Although we could not find putative T-DNA insertion lines for *OsbHLH058*, we found two T-DNA insertion rice lines for *OsbHLH059*. The insertion line 1B-08437, which we designated *b059*-*1*, possessed a T-DNA insertion in the 5′-untranslated region, and showed decreased *OsbHLH059* expression (Fig. S5b, c). The insertion line 3A-09300, which we designated b059OX-1, showed slightly enhanced *OsbHLH059* expression, presumably due to the insertion of enhancer sequences in the *OsbHLH059* promoter region adjacent to the *OsbHLH059* gene (Fig. S5b, c). When cultured hydroponically under Fe deficiency, the *b059*-*1* line showed a lower newest-leaf SPAD value than NT at days 2 and 3, whereas the b059OX-1 line showed a higher SPAD value than NT at days 1, 4, and 5 (Fig. 6a). The SPAD values of these lines settled nearer to the NT value at day 6 and thereafter (Fig. 6a). Notably, brownish or necrotic regions appeared in the newest leaves of the *b059*-*1* line, but not in those of NT or the b059OX-1 line, from day 5 of the Fe deficiency treatment (Fig. 6b, c). This symptom was not observed in any older leaves under Fe deficiency, or any leaves under Fe sufficiency. These results suggest that *OsbHLH059* expression levels also affect Fe nutrition in rice, in addition to *OsbHLH058*.

### Repression of *OsbHLH058* or *OsbHLH059* results in susceptibility to Fe deficiency and reduced expression of Fe-related genes

**Fig. 7:**
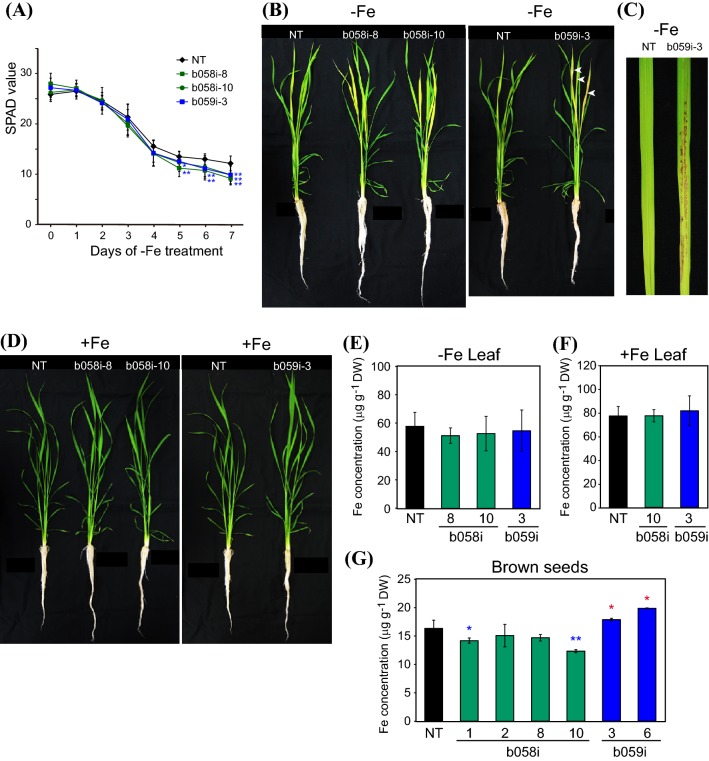
Fe deficiency tolerance of *OsbHLH058* and *OsbHLH059* knockdown plants produced by RNA interference. **a** Fe deficiency tolerance in hydroponic culture. Relative chlorophyll contents (SPAD values) of the newest leaves were measured after the onset of Fe deficiency treatment. **b** Representative plants on day 7 of Fe-deficient hydroponic culture. Three plants were bundled for culture of each line. White arrowheads indicate representative positions of brown spots in the newest leaves. **c** Representative newest leaves on day 7 of Fe-deficient hydroponic culture. **d** Representative plants on day 7 of Fe-sufficient hydroponic culture. Three plants were bundled for culture of each line. **e**, **f** Fe concentration in leaf blades after Fe-deficient (**e**) or -sufficient (**f**) hydroponic culture for 7 d. **g** Fe concentration in brown seeds after pot culture in Fe-sufficient soil. NT, non-transformant; b058i, *OsbHLH058* knockdown lines; b059i, *OsbHLH059* knockdown lines produced by RNA interference. Means ± SD are shown (**a** n = 12 for NT and b059i-3, n = 6 for b058i-8, n = 9 for b058i-10; **e** n = 4 for NT and b059i-3, n = 2 for b058i-8, n = 5 for b058i-10; **f** n = 3 for NT and b058i-10, n = 4 for b059i-3; **g**: n = 7 or NT, n = 3 for b058i-1, 8 and 10, n = 4 for b058i-2, n = 2 for b059i-3 and 6). Asterisks indicate significant differences compared to NT at each time point and condition (two-sample Student’s *t*-test; *P < 0.05; **P < 0.01)

To further investigate the roles of *OsbHLH058* and *OsbHLH059* in Fe deficiency responses, we produced transgenic rice lines with repressed expression of *OsbHLH058* (designated b058i lines) or *OsbHLH059* (b059i lines) using the RNA interference method. We selected representative lines with decreased expression of *OsbHLH058* for b058i or *OsbHLH059* for b059i (Fig. S5d). We performed three separate hydroponic experiments to impose Fe deficiency on these lines, as compared with NT (Figs. 7, S6). All selected b058i and b059i lines showed susceptibility to low Fe availability, as indicated by lower newest-leaf SPAD values than NT after the progression of Fe deficiency at around day 5 and thereafter (Figs. 7a, S6a). These lines had yellower leaves than NT at day 7 (Fig. 7b). Moreover, b059i lines, but not b058i lines or NT, exhibited severe browning or necrosis in partial regions of the newest and the second newest leaves at day 6 and thereafter of Fe deficiency treatment, but not in older leaves (Fig. 7b, c, S6b, c), as was observed in the *b059*-*1* line (Fig. 6b, c). The b058i and b059i lines showed similar growth to NT, with no visible phenotypes, under Fe sufficiency (Fig. 7d).

Leaf Fe concentrations among these lines after hydroponic culture were similar to NT levels under either Fe-deficient or -sufficient conditions (Fig. 7e, f). In brown seeds, b058i lines 1 and 10 contained lower concentrations of Fe, whereas b059i lines 3 and 6 showed higher Fe concentrations, compared with NT (Fig. 7g). We also tried to dissect Fe localization in the newest leaves of Fe-deficienct and -sufficient plants by Perls DAB staining (Roschzttardtz et al. 2009), but no clear differences between the genotypes were observed (data not shown).

**Fig. 8:**
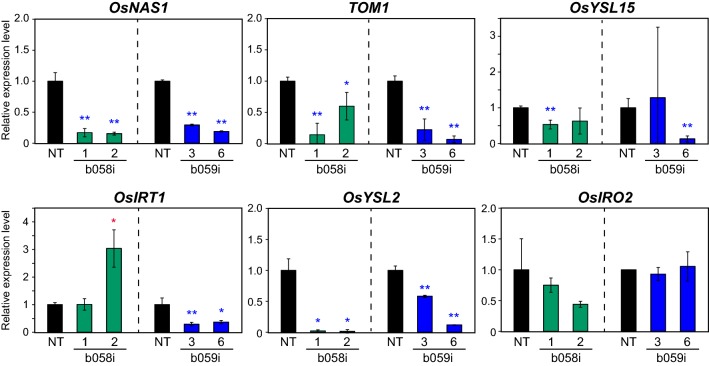
Expression levels of representative Fe-related genes in *OsbHLH058* and *OsbHLH059* knockdown plant roots. Transcript levels in Fe-sufficient roots of *OsbHLH058* knockdown (b058i) lines 1 and 2 and NT after 8 days of hydroponic culture, and in roots of *OsbHLH059* knockdown (b059i) lines 3 and 6 and NT lines after 9 days of hydroponic culture, were quantified by RT-PCR. Transcript abundance was normalized against the rice α-2 tubulin transcript level and expressed as a ratio relative to NT root levels in each experiment. Means ± SD (n = 3) are shown. Asterisks indicate significant differences from the NT value at each condition (two-sample Student’s *t*-test; *P < 0.05; **P < 0.01)

We then investigated the expression levels of the Fe-related genes *OsNAS1*, *TOM1*, *OsYSL15*, *OsIRT1, OsYSL2*, and *OsIRO2* in roots of the b058i and b059i lines and NT hydroponically grown under Fe sufficiency (Fig. 8). In b058i lines 1 and 2, *OsNAS1*, *TOM1*, and *OsYSL2* expression was strongly repressed compared with NT. *OsYSL15* and *OsIRO2* expression also tended to be repressed in these lines, whereas that of *OsIRT1* was not repressed but, rather, induced, in b058i line 2. In b059i lines 3 and 6, *OsNAS1*, *TOM1*, *OsIRT1*, and *OsYSL2* expression was strongly repressed compared with NT. *OsYSL15* expression was repressed only in line 6, whereas *OsIRO2* expression levels were similar between the b059i lines and NT.

We also examined the expression of these genes in leaves of the identical plants (Fig. S7). *TOM1* and *OsYSL15* expression was not detected in Fe-sufficient leaves. The b058i lines showed repressed expression of *OsYSL2* and *OsIRO2*, but not *OsNAS1* and *OsIRT1*, compared with NT. The b059i line 6 showed repressed expression of *OsYSL2* and tendency of reduced expression of *OsNAS1*, while the b059i line 3 did not show repressed expression but rather showed increased expression of *OsIRT1*, *OsYSL2* and *OsIRO2*, compared with NT.

Collectively, these results indicate that both OsbHLH058 and OsbHLH059 positively regulate these Fe-related genes in a similar but not identical manner.

## Discussion

### OsbHLH058 positively regulates Fe deficiency responses presumably downstream of OsHRZs

In the present study, we investigated the roles of OsbHLH058 and OsbHLH059 in comparison with those of other members of subgroup IVc bHLH TFs in rice: OsbHLH057 and OsbHLH060. We observed clear differences in expression patterns among these four genes (Fig. 1, Table S2). *OsbHLH058* expression was markedly repressed under Fe deficiency in roots and leaves. *OsbHLH059* expression also tended to be repressed in some tissues, whereas *OsbHLH057* expression was significantly induced in roots and leaves. We also detected slight *OsbHLH060* induction in roots and leaves, whereas our previous microarray results (Ogo et al. 2008) indicated subtle but statistically insignificant induction of *OsbHLH060* in roots and leaves (Table S2). Zhang et al. (2017) reported no induction of *OsbHLH060* under Fe deficiency. Notwithstanding slight differences in these results, possibly due to growth conditions or cultivars, these results suggest that *OsbHLH060* transcript levels are not much affected by Fe nutritional conditions. Each of the four *IVc bHLH* genes also showed distinct diurnal and jasmonate-responsive expression patterns based on microarray results, which may underlie functional diversification within this subgroup.

Our yeast two-hybrid assay showed clear interaction of OsbHLH058, but not OsbHLH059, with OsHRZ1 and OsHRZ2 (Fig. 2). Domain deletion analysis suggested that these interactions require the rubredoxin-type fold and the CHY or CTCHY Zn-finger or their N-terminal flanking region (Fig. 2). The functions of these domains have not yet been determined, but structural analysis of the human Pirh2 protein, which shares these domains and the RING Zn-finger domain with HRZs/BTS (Matthiadis and Long 2016), has indicated that these domains in Pirh2 bind Zn to form tertiary structure, which is presumably essential for interaction with other proteins (Sheng et al. 2008). Because HRZs are thought to bind both Fe and Zn via these domains (Kobayashi et al. 2013), intracellular metal status may influence the tertiary structure around these HRZ domains to alter their affinity to interacting partners, including OsbHLH058. Under our assay conditions, OsbHLH060 also showed interaction with OsHRZ1 and OsHRZ2, with similar domain requirements; however, this interaction was observed only in low-stringency medium, suggesting that the interaction with HRZs is weaker in OsbHLH060. Zhang et al. (2017) reported interaction between OsbHLH060 and a C-terminal portion of OsHRZ1 including the RING Zn-finger domain, in a high-stringency medium. Differences in the partial sequences of OsHRZ1 used in these two experiments may explain differences in interaction strength observed between OsbHLH060 and OsHRZ1. We could not include the RING Zn-finger domain in a HRZ bait due to strong auto-activation (data not shown), although Zhang et al. (2017) did not report such auto-activation.

The interaction of OsbHLH058 with HRZ ubiquitin ligases suggests possible ubiquitination of the former by the latter. Indeed, Zhang et al. (2017) reported that OsbHLH060 was ubiquitinated by OsHRZ1, based on in vitro ubiquitination and a degradation assay. Therefore, we used OsbHLH058 and OsbHLH060 as potential targets in our in vitro ubiquitination assay. However, we did not detect ubiquitination of these bHLH proteins by either OsHRZ1 or OsHRZ2 (Fig. 3). We also did not detect ubiquitination of OsbHLH059 by OsHRZ1 (Fig. 3). These bHLH proteins formed high-molecular-weight smear bands, even with inactive E3 controls (Fig. 3); this type of negative control experiment was not reported in Zhang et al. (2017). Similarly, no ubiquitination reaction of AtbHLH105, AtbHLH115, VOZ1, and VOZ2 was reported in *Arabidopsis* BTS in two recent studies (Selote et al. 2015, 2018). Collectively, no concrete evidence of HRZs/BTS ubiquitination targets has been reported to date, although subgroup IVc bHLH TFs are promising candidates in both rice and *Arabidopsis*. Very recently, FIT, a subgroup IIIa bHLH TF, was shown to be an ubiquitination target of *Arabidopsis* BTSL2 by in vitro ubiquitination assay, but this assay also lacked inactive E3 controls (Rodríguez-Celma et al. 2019b).

Although the physiological relevance of interaction between OsbHLH058 and HRZs is unclear, analysis of transgenic lines with overexpressed or repressed expression of *OsbHLH058* clearly showed its involvement in Fe deficiency responses (Figs. 4, 5, 7, 8, S5, S6). *OsbHLH058* overexpression lines showed enhanced tolerance to Fe deficiency, accumulation of higher Fe concentrations in seeds and Fe-sufficient leaves (Fig. 4), and induced expression of various Fe deficiency-inducible genes for Fe uptake and translocation in Fe-sufficient roots (Fig. 5a). These results suggest that OsbHLH058 positively regulates these genes to facilitate Fe uptake and translocation to shoots and seeds. This hypothesis is further supported by basically the opposite phenotype of the *OsbHLH058* knockdown lines, including susceptibility to Fe deficiency, lower Fe concentrations in seeds (Figs. 7, S6), and repressed expression of various Fe deficiency-inducible genes examined in Fe-sufficient roots and leaves (Fig. 8, S7). Fe concentration in Fe-sufficient leaves and *OsIRT1* expression in roots did not show a clear and opposite change from that of the overexpression lines, perhaps due to possible complementation of *OsbHLH058* knockdown by other bHLH genes, or secondary effects of *OsbHLH058* overexpression. Fe deficiency-inducible Fe-related genes in rice can be categorized into four groups according to their functions and expressional patterns in the regulatory network: (i) genes for Fe(III)-DMA uptake and translocation (including *OsNAS1*, *TOM1*, and *OsYSL15*); (ii) genes for Fe^2+^ uptake (including *OsIRT1*); (iii) genes for internal Fe translocation (including *OsYSL2*); (iv) genes regulating group (i)–(iii) genes [including *OsIRO2*, which positively regulates group (i) genes] (Kobayashi et al. 2014). The expression patterns of *OsbHLH058* overexpression and knockdown lines (Figs. 5a, 8, S7) suggest that OsbHLH058 positively regulates all four groups, among which group (i) and (iii) genes may be more strongly regulated by OsbHLH058 in Fe-sufficient roots.

Interestingly, enhanced expression of these Fe-related genes in *OsbHLH058* overexpression lines was evident only under Fe sufficiency, but was diminished or even reversed to lower expression levels than in NT under Fe deficiency, except for *OsYSL2* (Fig. 5a vs. b). This pattern of expressional change is strikingly similar to that observed in *HRZ* knockdown lines, including extremely high *OsYSL2* induction, even under Fe deficiency (Kobayashi et al. 2013). This similarity, along with the interaction of OsbHLH058 and HRZs, suggests that OsbHLH058 function may be regulated by, or linked to, HRZs. Enhanced expression of Fe-related genes in *HRZ* knockdown lines is still more prominent under Fe excess conditions (Aung et al. 2018), suggesting that HRZ function is regulated by Fe abundance, which may account for intracellular Fe sensing. OsbHLH058 may be an output of an HRZ-mediated Fe sensing mechanism, in which the function of HRZs may be partially mediated by inhibition of OsbHLH058. Although its molecular mechanism remains completely unknown, binding to HRZs may inhibit homo- or heterodimer formation of OsbHLH058 for its proper function.

### The role of OsbHLH059 in positive regulation of Fe deficiency responses is similar to but distinct from that of OsbHLH058

Although OsbHLH059 did not show interaction with HRZs or detectable ubiquitination by OsHRZ1 (Figs. 2, 3), analysis of the transgenic lines showed that OsbHLH059 is also involved in Fe deficiency responses. The *OsbHLH059* overexpression line with enhancer-tagged T-DNA showed slight tolerance to Fe deficiency (Fig. 6a), although its phenotype was not strong, possibly due to a very weak increase in *OsbHLH058* expression (Fig. S5). Clearer results were obtained from *OsbHLH059* knockdown lines (Figs. 6, 7, 8, S5, S6, S7). Similar to *OsbHLH058*, *OsbHLH059* knockdown lines showed susceptibility to Fe deficiency and repressed expression of various Fe-related genes in roots (Figs. 6, 7, 8, S6). In addition, *OsbHLH059* knockdown lines by either T-DNA insertion or RNA interference exhibited characteristic brownish or necrotic regions in new leaves after prolonged Fe deficiency treatment (Figs. 6, 7, S5), which were never observed in NT or *OsbHLH058* knockdown lines. Because this phenotype was restricted to severely chlorotic regions suffering from Fe deficiency, this result may be due to local leaf damage from either extremely low Fe abundance or ectopic accumulation of other metals. Although we detected no decrease in Fe concentrations in whole leaves of the *OsbHLH059* knockdown lines (Fig. 7e), some impairment of Fe distribution in restricted leaf areas may have occurred due to *OsbHLH059* knockdown. We did not observe clear differences in Fe localization in the newest leaves by Perls DAB staining (data not shown), perhaps because of very low Fe concentration. A similar occurrence of brown spots in Fe-deficient leaves was reported for *OsbHLH060* knockout lines *pri1*-*1* and *pri1*-*2* (Zhang et al. 2017); however, this phenomenon has not been investigated further.

Another distinguishable difference between *OsbHLH059* and *OsbHLH058* knockdown lines was observed in seed Fe concentration; *OsbHLH059* knockdown lines showed higher Fe concentrations than NT, in contrast to *OsbHLH058* knockdown lines (Fig. 7g). Expression patterns of Fe-related genes were also different between *OsbHLH059* and *OsbHLH058* knockdown lines (Figs. 8, S7). These results indicate that OsbHLH059 enhances the pathway of Fe deficiency responses for proper Fe uptake and distribution to rice tissues, in a manner similar to but distinct from that of OsbHLH058. Based on our gene expression results (Figs. 8, S7), OsbHLH059 may positively regulate a large subset of Fe-related genes in Fe-sufficient roots, including those for (i) genes for Fe(III)-DMA uptake and translocation, (ii) genes for Fe^2+^ uptake, and (iii) genes for internal Fe translocation, but not (iv) *OsIRO2*. In Fe-sufficient leaves, *OsbHLH059* knockdown lines did not show repression of Fe-related genes except for *OsYSL2* in b059i line 6 (Fig. S7). Instead, enhanced expression of *OsIRT1*, *OsYSL2* or *OsIRO2* was observed in leaves of one or both b059i lines analyzed (Fig. S7), possibly due to secondary effects of disturbed Fe availability, or possible negative regulation by OsbHLH059, especially for *OsIRO2* which showed much enhanced expression in both b059i lines (Fig. S7).

In contrast to OsbHLH059, OsbHLH060 appears to positively regulate many Fe-related genes representative of all groups (i)–(iv) under both Fe sufficiency and deficiency, based on the expression patterns of *OsbHLH060* knockdown lines in roots (Zhang et al. 2017). Together, these results suggest that three members of the subgroup IVc bHLH TFs, OsbHLH058, OsbHLH059, and OsbHLH060, regulate largely overlapping, but not identical, subsets of Fe deficiency-inducible genes (Fig. S1b).

In *OsbHLH058* and *OsbHLH059* knockdown roots, the expression levels of many target genes such as *OsNAS1*, *TOM1*, and *OsYSL2* often decreased to less than 50% of each NT control (Fig. 8), irrespective of high sequence and functional similarity with subgroup IVc bHLH TFs. This result suggests that the expression of these target genes requires both OsbHLH058 and OsbHLH059 in a synergistic, rather than additive, fashion. Although we did not detect interaction between OsbHLH059 and HRZs (Fig. 2), a possible synergistic effect between OsbHLH059 and OsbHLH058 suggests indirect involvement of OsbHLH059 in the HRZ pathway.

Such transactivation function of OsbHLH058 and OsbHLH059 could be mediated by heterodimer formation or synergistic effects of plural *cis*-acting elements in target genes. Many bHLH TFs preferentially recognize E-box (CANNTG) or G-box (CACGTG) sequences; sequence recognition preferences can be predicted from amino acid sequences of specific residues in the basic region (Toledo-Ortiz et al. 2003). Based on this criterion, all subgroup IVc bHLH TFs in rice and *Arabidopsis* are predicted to bind preferentially to G-box (Toledo-Ortiz et al. 2003), suggesting significant sharing of *cis*-acting sequences among the subgroup, with heterodimer formation possibly playing important roles. To discover the precise functions of the apparently diversified bHLH TFs related to Fe homeostasis would require precise and comprehensive characterization of the target genes of each member, including the distribution of corresponding *cis*-acting elements in each gene promoter context, homo/heterodimer formations of each member, the functional significance of these homo/heterodimer formations, and clarification of the possible involvement of HRZs/BTS in the abundance and activity of each bHLH member. The clarification of these issues would be key to understanding overall regulation cascades in plant Fe deficiency responses.

## Electronic supplementary material

Below is the link to the electronic supplementary material.

Supplementary material 1 (DOCX 1068 kb)
