# Supplementary material for: OsbHLH058 and OsbHLH059 transcription factors positively regulate iron deficiency responses in rice
Source: Plant Mol Biol. 2019 Sep 24;101(4):471–86. doi: 10.1007/s11103-019-00917-8 (PMC6814640; doi:10.1007/s11103-019-00917-8)
Supplement: Supplementary file 1 — Supplementary material 1 (DOCX 1068 kb) [file 11103_2019_917_MOESM1_ESM.docx]

**Supplemental Tables**

**Table S1.** Sequences of primers used in all experiments.

**Table S2.** Expression analysis of *subgroup IVc bHLH* genes using 44K microarray data.

Expression ratios of *OsbHLH057*, *OsbHLH058*, *OsbHLH059*, and *OsbHLH060* were examined by previous rice 44K microarray experiments as follows. –Fe response: Fe deficiency responses in wild-type rice (Ogo et al. 2008). –Fe/+Fe: expression ratio calculated as (Fe deficiency treatment for 7 days)/(Fe sufficiency treatment for 7 days). –Fe response (microdissection): Fe deficiency responses in wild-type microdissected tissues (Ogo et al. 2014). NewDC: bases of new leaves at the lowest node of the shoot. oldDC: bases of old leaves at the lowest node of the shoot. VB: root vascular bundle. Cor: root cortex. EP: root epidermis and exodermis. HRZ dependence: expression ratios of *HRZ*-knockdown lines/non-transformants (i/NT) at day 7 of Fe sufficiency (+Fe 7 d) or day 1 or 7 of deficiency (–Fe 1d, 7d) based on previous microarray analysis (Kobayashi et al. 2013, as calculated in Kobayashi et al. 2016). IDEF1 dependence: based on previous microarray analysis (Kobayashi et al. 2009). IN/NT: expression ratio calculated as *IDEF1* induction line/non-transformant. i/NT: expression ratio calculated as *IDEF1*-knockdown line/NT. IDEF2 dependence: based on previous microarray analysis (Ogo et al. 2008). i/NT: expression ratio calculated as *IDEF2*-knockdown line/non-transformant. –Fe: Fe deficiency for 7 days. +Fe: Fe sufficiency for 7 days. R: roots. S: shoots. Ratios with Agilent’s *P*-value < 0.05 (two-sample Student’s *t*-test) are indicated in bold, except for microdissection analysis by Ogo et al. (2014).

**Supplemental Figures**

**Figure S1**

**Fig. S1** Simplified summary of the rice regulatory pathway of HRZ/BTS → IVc bHLHs → Ib/IVb bHLHs → Fe deficiency-inducible genes. **a** Present knowledge. Functions of OsbHLH057, OsbHLH058, and OsbHLH059, have yet to be investigated, and thus these factors are shown in gray. **b** Advanced version reflecting the results obtained in the present research. Black lines, transcriptional regulation; red lines, protein-level regulation (two-way arrows represent protein–protein interactions); blue lines, unknown types of regulation. Broken lines indicate putative pathways.

**Figure S2**


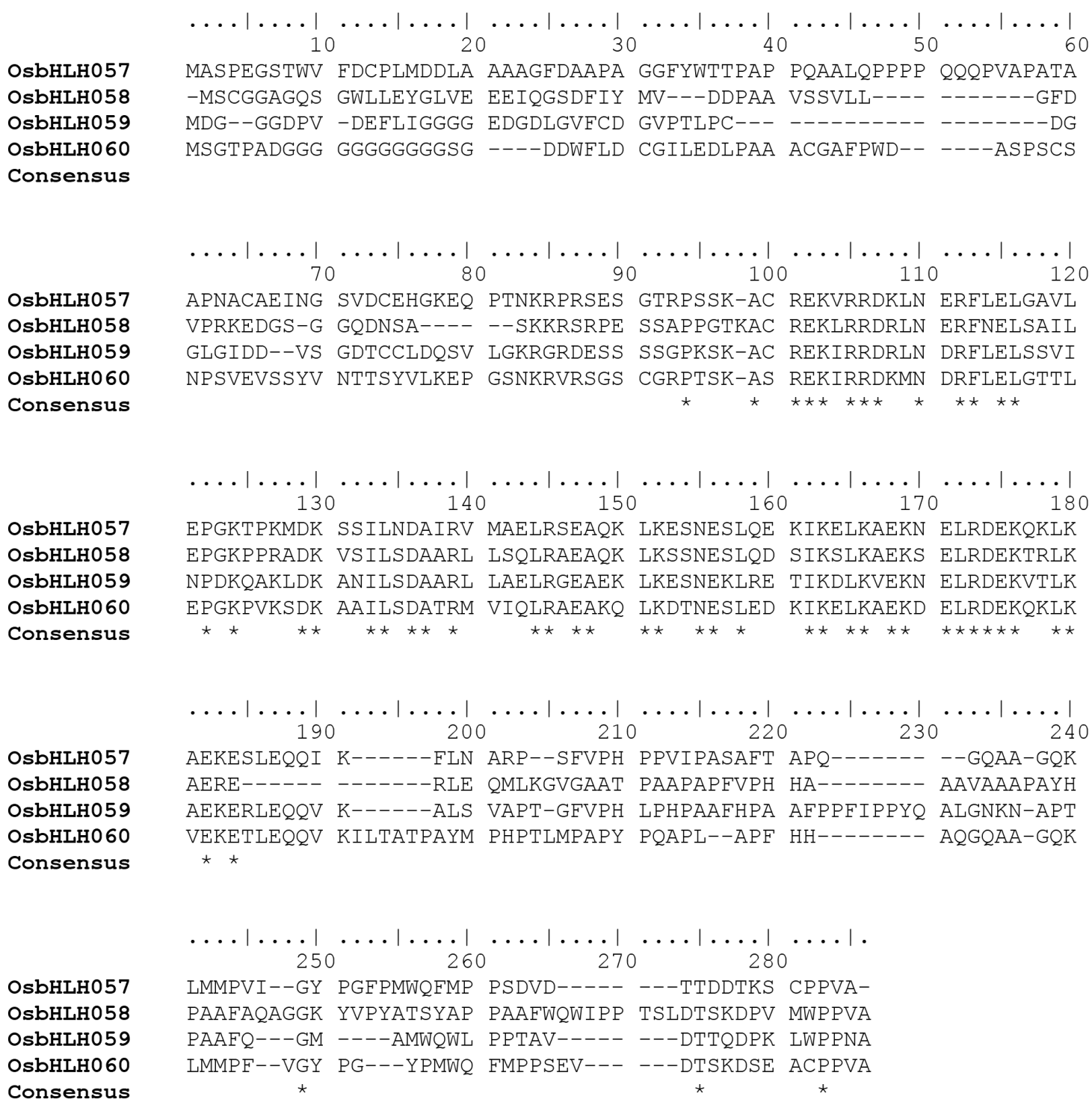


**Fig. S2** Sequence alignment of the four rice subgroup IVc bHLH proteins (OsbHLH057, OsbHLH058, OsbHLH059, and OsbHLH060).

**Figure S3**


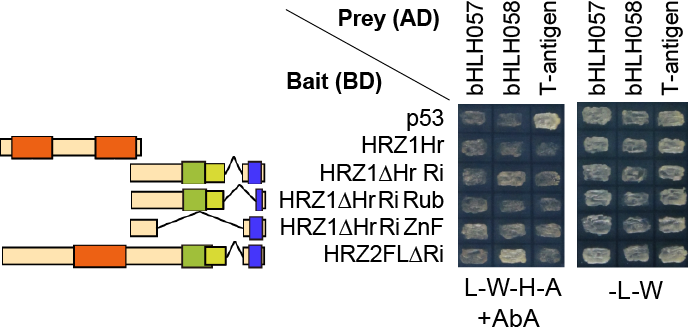


**Fig. S3** Additional yeast two-hybrid analysis of OsHRZ and subgroup IVc bHLH proteins. Yeasts were transformed with GAL4 DNA-binding domain (BD)-fused bait and GAL4 activation domain (AD)-fused prey and cultured for 5 days. Interaction was detected by enhanced growth in high-stringency selective medium (-L-W-H-A+AbA). Basal growth was confirmed in basal medium (-L-W). BD-p53 and AD-large T antigen (T-antigen) are controls provided by the manufacturer.

**Figure S4**


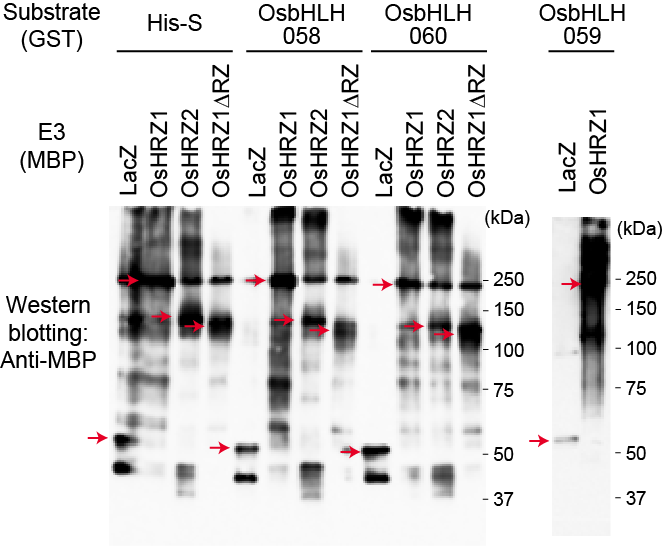


**Fig. S4** Confirmation of E3 ligase activity in an *in vitro* ubiquitination assay. Blots used for analysis shown in Fig. 3 were re-probed using anti-MBP antibody. The positions and sizes of molecular mass markers are shown to the right of each blot. Monomeric free MBP-fused proteins are indicated with arrows.

**Figure S5**


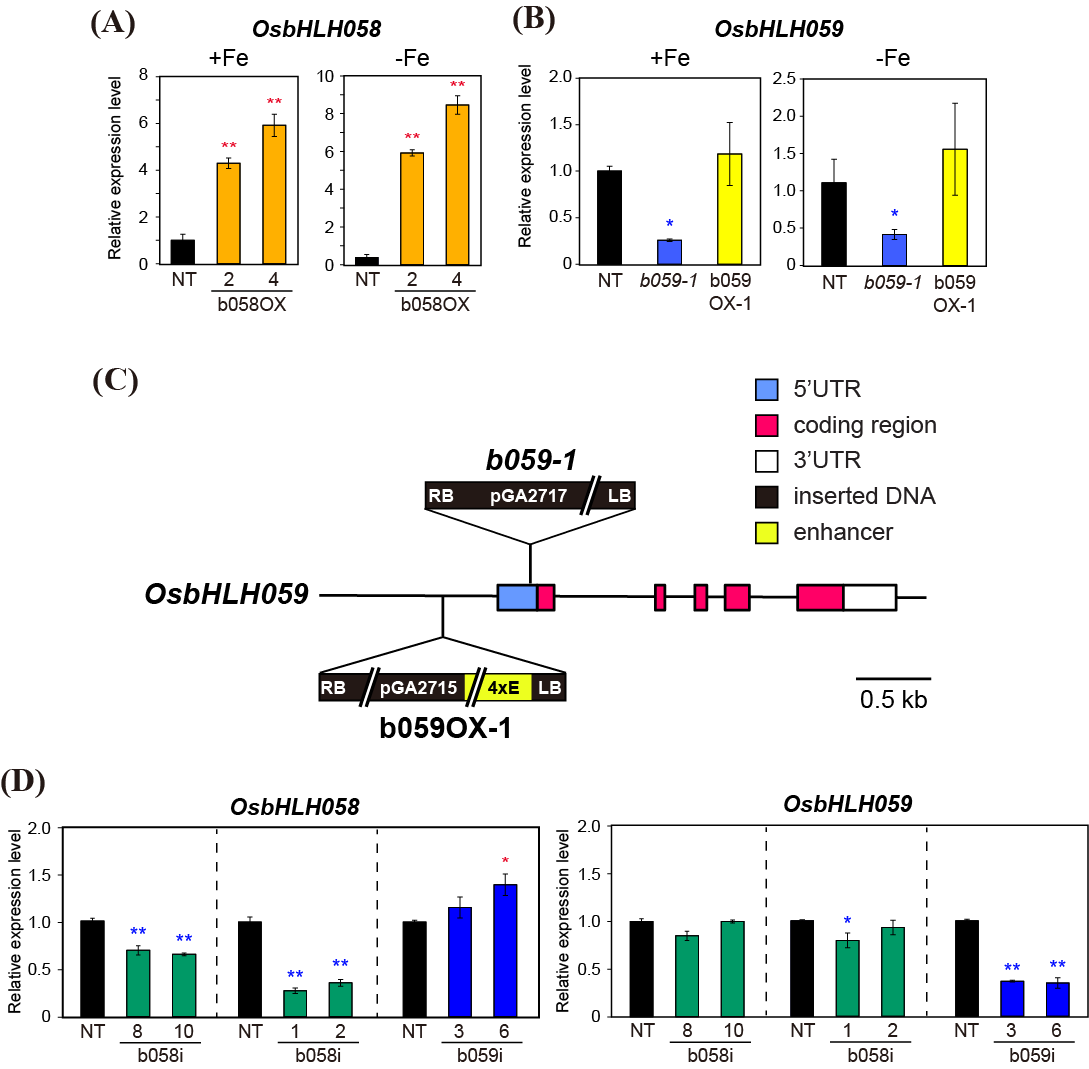


**Fig. S5** Expression levels of *OsbHLH058* and *OsbHLH059* in transformants (**a, b, d**), and positions of T-DNA insertions in *OsbHLH059* (**c**). Transcript levels of *OsbHLH058* and *OsbHLH059* were quantified by RT-PCR in roots cultured under Fe sufficiency (+Fe) or deficiency (–Fe) in transformants compared with non-transformants (NT). **a** *OsbHLH058* expression in roots of *OsbHLH058* overexpression (b058OX) lines 2 and 4 and NT, after 7 days of hydroponic culture under Fe sufficiency or deficiency. **b** *OsbHLH059* expression in roots of the *OsbHLH059* knockdown line by T-DNA insertion (*b059-1*), the *OsbHLH059* overexpression line by T-DNA insertion (b059OX-1), and NT, after 9 days of hydroponic culture under Fe sufficiency or deficiency. **c** Genome structure of *OsbHLH059* and insertion patterns of T-DNA in the *b059-1* and b059OX-1 lines. LB, left border; RB, right border; UTR, untranslated region; 4xE, quadruple enhancer sequence. **d** *OsbHLH058* and *OsbHLH059* expression in Fe-sufficient roots of *OsbHLH058* knockdown (b058i) lines 8 and 10 and NT after 7 days of hydroponic culture, b058i lines 1 and 2 and NT after 8 days of hydroponic culture, and RNA interference *OsbHLH059* knockdown (b059i) lines 3 and 6 and NT after 9 days of hydroponic culture, under Fe sufficiency. Transcript abundance was normalized against the rice α-2 tubulin transcript level and expressed as a ratio relative to levels in +Fe NT roots in each experiment. Means ± SD (n = 3) are shown. Asterisks indicate significant differences from the NT value at each condition (two-sample Student’s *t*-test; *, *P* < 0.05; **, *P* < 0.01).

**Figure S6**

**
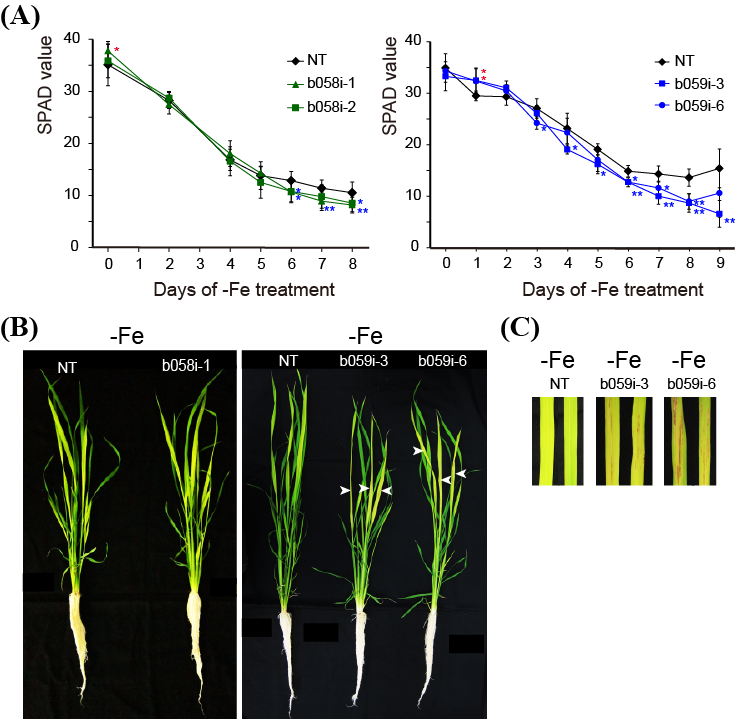
**

**Fig. S6** Additional hydroponic experiments for *OsbHLH058* and *OsbHLH059* knockdown plants produced by RNA interference. **a** Fe deficiency tolerance in hydroponic culture. Relative chlorophyll contents (SPAD values) of the newest leaves were measured after the onset of Fe deficiency treatment. **b** Representative plants on day 8 of Fe-deficient hydroponic culture for *OsbHLH058* knockdown (b058i) line 1 compared with non-transformants (NT), or day 9 of Fe-deficient hydroponic culture for *OsbHLH059* knockdown (b059i) lines 3 and 6 compared with NT. Three plants were bundled for culture of each line. White arrowheads indicate representative positions of brown spots in the newest leaves. **c** Representative newest leaves on day 9 of Fe-deficient hydroponic culture.

**Figure S7**

**
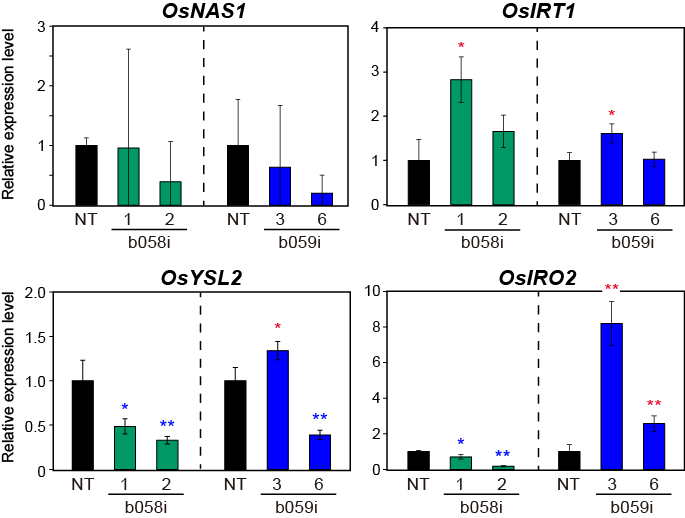
**

**Fig. S7** Expression levels of representative Fe-related genes in *OsbHLH058* and *OsbHLH059* knockdown plant leaves. Transcript levels in Fe-sufficient leaves of *OsbHLH058* knockdown (b058i) lines 1 and 2 and NT after 8 days of hydroponic culture, and in roots of *OsbHLH059* knockdown (b059i) lines 3 and 6 and NT lines after 9 days of hydroponic culture, were quantified by RT-PCR. *TOM1* and *OsYSL15* expression was not detected in these samples. Transcript abundance was normalized against the rice α-2 tubulin transcript level and expressed as a ratio relative to NT root levels in each experiment. Means ± SD (n = 3) are shown. Asterisks indicate significant differences from the NT value at each condition (two-sample Student’s *t*-test; *, *P* < 0.05; **, *P* < 0.01).
